# Editorial: Women in nutrition and brain health

**DOI:** 10.3389/fnut.2023.1229751

**Published:** 2023-09-13

**Authors:** Eef Hogervorst, Crystal Haskell-Ramsay, Nafisa M. Jadavji

**Affiliations:** ^1^National Centre for Sport and Exercise Medicine (NCSEM), Loughborough University, Loughborough, United Kingdom; ^2^Department of Psychology, Northumbria University, Newcastle upon Tyne, United Kingdom; ^3^Department of Biomedical Sciences, College of Graduate Studies, College of Veterinary Medicine, College of Osteopathic Medicine, Midwestern University, Glendale, AZ, United States; ^4^Department of Child Health, College of Medicine Phoenix, University of Arizona, Phoenix, AZ, United States; ^5^Department of Neuroscience, Carleton University, Ottawa, ON, Canada

**Keywords:** women, STEM—Science Technology Engineering Mathematics, academic research, one-carbon metabolism, health

Representation of women in academic research is not well-balanced and women hold the majority of non-tenure-track lecturer- and instructor positions. There are fewer women than men in higher academic and managerial positions. Furthermore, fewer than 30% of researchers worldwide are women (1). The leaky pipeline, long-standing biases and gender stereotypes are discouraging girls and women away from science-related fields and STEM research (2–4). Science and gender equality are, however, essential to ensure sustainable development as highlighted by UNESCO (1). Health inequality is also often driven by socioeconomic inequality, and these have a reciprocal relationship, with increasing dependency forcing fewer people to contribute financially. This is especially stark in low- and middle-income countries. We recently suggested that continued education in girls, but also in single older women living in impoverished rural areas in Indonesia is crucial to help sustain them as they age, so that they can live independent and healthy lives (5).

To change traditional mindsets, gender equality must be promoted, stereotypes defeated, and girls and women should be encouraged to pursue STEM careers. The work presented in this Research Topic highlights the diversity of research performed across the entire breadth of Nutrition and Brain Health research and presents advances in theory, experiment, and methodology with applications to compelling problems. All research published in this topic shares work conducted by women and that is important for women.

A study by Bathalha et al. investigated the association between mental health during pregnancy and postpartum vs. vitamin B12 status in serum and milk, as well as levels of homocysteine. These toxic amino acids are components of one-carbon metabolism and play a vital role in brain function of both the mother and baby. Researchers found that women with anxiety symptoms in the third trimester of pregnancy presented with higher serum homocysteine during the first week postpartum and had lower levels of vitamin B12. Serum levels of vitamin B12 also correlated with milk levels. This study suggests that monitoring maternal mental health and biomarkers, such as vitamin B12 and homocysteine, could be effective in managing maternal and child nutritional status and are associated with mental health in mothers which could affect child development. High homocysteine and low B12 and folate status are important risk factors over the lifespan and have also been found to be predictive for dementia and cardiovascular disease in later life (6). Alzheimer's disease, the most prevalent type of dementia, is more common in older women and has high human and economic costs. Alternatively, anxiety and poor nutritional status including milk production could be driven by other factors, such as socioeconomic uncertainty, which situation could be exacerbated by childbirth. However, a good nutritional status including vegetable and fruit consumption could overcome detrimental effects of low socioeconomic status on mental health, including anxiety (7) and cognitive function in later life (8).

Another paper published in the field of one-carbon metabolism by Zhang et al. attempted to determine whether folic acid prior to radiation treatment impacted ovarian damage using a mouse model. Researchers administered folic acid daily for 3 weeks prior to radiation. In female mice given folic acid, they reported reduced levels of oxidative stress, inflammation, and enhanced DNA repair, as well as higher pregnancy rates and greater litter sizes, when compared to controls. This study demonstrated the protective effects of folic acid against radiation exposure in female mice. It suggests that prenatal exposure to folic acid, as a supplement and/or via nutrition (including dark green leafy vegetables and citrus fruits, for instance) could combat potential adverse effects on female reproductive capacity and health. Folic acid is one of the most potent nutritional factors to reduce high homocysteine (6).

Early nutrition plays an important role in the development and future health outcomes of an infant. However, the mechanisms through which this occurs remain elusive. We gained further insight into these possible mechanisms through a study by Charton et al.. The authors investigated the effects of human or cow's milk based infant formula vs. gut microbiota-brain-axis development. Using a pig model system, the researchers found that human milk induced different colonic and fecal microbiota profile, which impacted the gut permeability, as well as affected gene expression related to the immune response, epithelial barrier, endocrine function, nutrient transporters, and tryptophan metabolism. Gene expression in brain tissue was also measured, researchers found that genes encoding blood-brain barrier, endocrine function and short chain fatty-acid receptors were changed. These changes were predominantly found in the hypothalamic and striatal areas. This study used an integrative approach to better understand the influence of infant diet on the development of the microbiota-gut-brain axis. The gut-brain axis is increasingly recognized as an important driver of brain health over the lifespan.

The areca nut is classified as a carcinogen and used as an addictive substance, Hsu et al. investigated the qualitative effects of areca nut dependence in a Taiwanese population. The researchers examined correlations among areca nut self-awareness, nut cessation, and dependence based on various demographic variables. The researchers were not able to confirm previous results which reported that areca nut self-awareness affects areca refusal self-efficacy and that increased awareness aids in areca nut cessation (9). They report that areca nut self-awareness revealed significant correlations with age, occupation, and family support. The results of the study can be used to help implement areca nut cessation policies in the future. Family support systems are important for child development, but also for later life health in general, especially for women as they age (6).

We took part in this Research Topic to profile women academic researchers in STEM and related fields. Women lifting other women is important and gravely needed. We hope that the future of academic research is inclusive through strong mentoring, changes in policies, and better recognition of women in academic research to help them achieve the positions they deserve in academia and leadership. Lifelong learning is especially important for women, their development And health and should be supported by governmental and local policies worldwide.

## Author contributions

All authors listed have made a substantial, direct, and intellectual contribution to the work and approved it for publication.
